# Increased attention allocation to stimuli reflecting end-states of compulsive behaviors among obsessive compulsive individuals

**DOI:** 10.1038/s41598-023-39459-x

**Published:** 2023-07-27

**Authors:** Dana Basel, Moriah Magen, Amit Lazarov

**Affiliations:** School of Psychological Sciences, Tel Aviv University, 69978 Tel Aviv, Israel

**Keywords:** Human behaviour, Obsessive compulsive disorder

## Abstract

Attentional research in OCD has focused solely on threat stimuli, assumed to provoke related obsessions and ensuing compulsions. OCD-related stimuli depicting the completion of compulsive acts (“end-states”) have yet to be examined. Past research also neglected to explore the reliability of tasks used. Here, attention allocation to both stimuli types was examined. Participants with high (HOC) and low (LOC) levels of obsessive–compulsive symptoms freely viewed three blocks of 30 two-by-two picture matrices, each including two OCD-related (cleaning\checking\ordering) and two neutral pictures, presented for eight seconds, while their gaze was recorded. Participants completed two task versions – one with traditional threat stimuli and one with novel stimuli signaling compulsions end-states. Only the end-state version yielded significant results, showing that HOC participants, compared to LOC participants, spent significantly more time fixating on OCD-related stimuli. Results remained significant after controlling for anxiety, stress, and depression. Task reliability was high. OCD-related stimuli signaling end-states of compulsive behavior should be incorporated in attentional research in OCD.

## Introduction

Obsessive compulsive disorder (OCD) is a chronic and debilitating disorder with a lifetime prevalence of 2 to 3%^1^. It is characterized by *obsessions* – recurrent persistent thoughts, impulses, or images that are experienced as intrusive and inappropriate, causing marked anxiety or distress; and by *compulsions –* repetitive behaviors or mental acts that the person feels driven to perform in response to an obsession, or according to rules that must be applied rigidly^2^. OCD is associated with significant impairments in various life domains, including work, home, relationships, and social functioning^1^. Similar impariments are also noted in individuals with high levels of OCD symptoms^3–5^, who are considered as being at increased risk for later development of clinical OCD^6^.

Cognitive models of the disorder posit that misinterpretation of benign or otherwise “normal” intrusive thoughts contribute to the development and maintenance of the disorder^7,8^. According to these models, a “normal” intrusive thought becomes an obsession when the individual interprets the occurrence or content of the intrusion as a sign of personal responsibility for causing or preventing harm to oneself or others^9^. These misinterpretations result in a range of outcomes, including preferential attention allocation to stimuli related to one’s obsessions^10^, which, in turn, may increase intrusions reoccurrence and maintain obsessional thoughts and beliefs, motivating compulsive behaviors^11,12^.

The aforementioned biased attention allocation may manifest in easier or faster detection of OCD-related cues and stimuli (i.e. vigilance). For example, an OCD patient with checking symptoms might more easily/rapidly detect a turned on stove within an array of other appliances. Alternatively, biased attention may also manifest in later more goal-directed attentional processes, such as greater sustained attention on OCD-relevant stimuli (attentional maintenance). Here, a patient with contamination symptoms might maintain visual attention on a seemingly dirty cup within an array of cleaned dishes. Importantly, these two attention biases, vigilance and maintenance, are not mutually exclusive, and may operate conjointly – a patient with OCD may display facilitated threat detection, followed by difficulty to disengage attention once threat has been detected^13^. Trying to ascertain these biased attentional processes in OCD, research employing eye-tracking methodology has been increasingly used^11,14–18^. In these studies, OCD and non-OCD participants are usually presented with OCD-related stimuli, coupled with neutral stimuli, while their gaze is continuously recorded. Different facets of eye-data (e.g. fixations) are then used to compare participants’ visual attention patterns.

To date, only a small number of eye-tracking-based attentional studies has been conducted in OCD, all using OCD-related threat stimuli (e.g. a dirty toilet) assumed to provoke corresponding obsessions (e.g. contamination obsessions) due to their threatening nature (for a review see^16^). Although evidence for vigilance is relatively limited^14,15^, most studies support attentional maintenance to OCD-related threat stimuli among obsessive–compulsive individuals^16^ (cf.^15^). Yet, while providing initial support for biased attention allocation in OCD, extant studies are characterized by two methodological shortcomings. First, most did not examine the psychometric properties of applied tasks and measures, with only one study addressing this important issue, but showing only moderate reliability^11^. Reliable tasks and measures are essential to inspire confidence in obtained results^19–24^, and is considered a major barrier in the advancement of attention research^21^. Second, most studies presented only two stimuli simultaneously^16^. However, more complex visual displays are needed to increase the generalizability of observed results to real-world settings, which usually involve more than two competing stimuli^25,26^. Moreover, eye-tracking measures are affected by stimuli array size, with different gaze pattern emerging when using simple vs. more complex visual displays^26,27^.

As noted above, all extant attentional studies in the field used OCD-related stimuli depicting situations aimed at provoking related obsessions^16^, echoing the phenomenology of OCD, according to which the disorder is characterized by obsession-related anxiety and/or distress^28^. Yet, obsession-related anxiety is often followed by the performance of corresponding compulsive behaviors, leading to relief and decreased distress, even if short lived^29^, reflective of negative-reinforcement processes^28^. Indeed, attentional research has shown increased attention allocation toward negatively-reinforced stimuli^30–36^. Thus, one intriguing question worth exploring is whether OCD-related stimuli depicting “end-states” of compulsive behavior (e.g. a perfectly clean and spotless sink) would result in attention allocation patterns similar to those noted for traditionally-used OCD-related threat stimuli^16^. As one’s environment is not exclusively comprised of clearly “threatening” cues, this important aspect needs to be examined. However, to date, no study has addressed this issue experimentally.

Here, we examined attention allocation patterns of participants with high and low levels of OCD symptoms to two types of stimuli, compared to neutral stimuli – traditional OCD-related threat stimuli, widely used in previous studies to provoke obsessions^16^, and novel stimuli depicting end-states of compulsive behaviors. To try and address the heterogeneity of OCD, stimuli included cleaning, checking, and ordering cues, similar to previous studies in the field^17,18^. In addition, addressing the aforementioned methodological limitations, we: (1) assessed the task’s reliability (i.e. internal consistency), across and within groups; and (2) used complex visual displays of four co-presented stimuli, two OCD-related and two neutrals. Based on past research in the field^16^, we expected that compared to non-OC participants, OC participants would show an attention allocation pattern favoring threat OCD-related stimuli, over neutral ones. As no study to date has explored attention allocation toward OCD-related stimuli depicting end-states of compulsive behaviors, we had no specific predictions for this stimuli type.

## Method

### Participants

Three hundred and thirty-seven students were screened using the Obsessive–Compulsive Inventory-Revised (OCI-R^37^) at the beginning of the academic year. Those scoring at the top of the OCI-R distribution comprised the high obsessive–compulsive (HOC) group, contingent on having a score > 27, which is above the clinical cutoff score on this scale (OCI-R = 21^37^), denoting severe OCD^38^. Only those scoring above the clinical cutoff score of 21 also on the day of their participation, held several weeks following the initial screening, were enrolled in the study. As score fluctuations between the two time points (the beginning of the academic school year and the day of participation, which may be held several weeks later) are quite possible, OCI-R score > 27 during the first time point increased the probability of OCI scores remaining above 21 in the second time-point. Out of those scoring above 27, only three potential participants were not enrolled in the study due to a drop in their OCI-R score on the day of their participation. The low obsessive–compulsive (LOC) group consisted of those scoring at the bottom of the distribution, contingent on having an OCI-R score < 10, as a score below 15 is considered as reflecting minimal obsessive–compulsive symptoms^38^. The final sample included 60 participants: Thirty in the HOC group (*M*age = 23.53 years, *SD* = 1.25, range = 21–26 years; 7 men), and 30 in the LOC group (*M*age = 23.77 years, *SD* = 2.09, range = 21–31 years; 9 men). Three LOC participants were excluded from analyses due to technical difficulties related to the eye-tracking apparatus, which precluded data collection. Participants provided informed consent and received course credit for participation.

The study protocol was approved by the Research Ethics Council of Tel Aviv University. We only invited participants with normal or corrected-to normal vision, excluding usage of multi-focal eyewear to prevent eye-tracking calibration difficulties.

### Measures

Participants were assessed for obsessive compulsive symptoms (OCI-R^37^), depression, stress, and anxiety (Depression, Anxiety and Stress Scales-21; DASS-21^39^).

#### Obsessive–compulsive symptoms

Obsessive–compulsive symptoms were measured using the OCI-R^37^, an 18-item self-report questionnaire assessing obsessive–compulsive symptoms. Participants indicate their level of distress associated with each symptom on a 5-point Likert scale ranging from 0 (not at all) to 4 (very much), resulting in a 0-to-72 total score. The OCI-R has been shown to have good validity, test–retest reliability and internal consistency in both clinical^37,40,41^ and non-clinical samples^42,43^. Internal consistency in the present study was 0.94.

#### Depression, anxiety, and stress symptoms

Depression, anxiety, and stress symptoms were measured by using the Depression, Anxiety and Stress Scales-21 (DASS-21^39^). The DASS-21 is a 21-item self-report questionnaire yielding three sub-scales of seven items each, assessing dimensional components of depression, anxiety and stress. Each individual item is rated on a 4-point scale ranging from 0 (the item does not apply to me at all) to 3 (the item applies to me very much or most of the time), on which participants indicate how much the statement applied to him/her experience over the past week. The DASS-21 has been shown to have high reliability, validity and internal consistency in both clinical and non-clinical groups^39,41,44–46^. Internal consistency in the present study was 0.93, 0.86, and 0.93, for the depression, anxiety and stress subscales, respectively.

### Attention allocation task

Attention allocation was assessed using a well-validated and widely used free-viewing eye-tracking task^13,19,25^ adapted for the purpose of the current study. The task was designed and executed using the Experiment Builder software (version 2.1.140; SR Research Ltd., Ottawa, Ontario, Canada). Following a previous study employing a similar attention allocation assessment task and approach^47,48^, we included three different blocks, each focusing on a different prominent OCD theme^49^ – a checking block, a cleaning block, and an ordering/symmetry block – delivered in a counterbalanced manner within each group. For each block, 12 OCD-related and 12 neutral chromatic pictures were used, from which 30 different 2-by-2 matrices were prepared. Each stimulus extends 255-by-225 pixels, including a 10-pixel white margin frame, for an overall matrix size of 550-by-550 pixels. Each single picture appeared 5 times per block. Single pictures appeared randomly at any position within the matrix while ensuring that each picture appeared only once in a given matrix. Different neutral pictures were used across the three blocks to eliminate familiarity effects.

Each trial began with a centrally presented fixation-cross mandating a 1-s fixation for the matrix itself to appear. Then the matrix appeared for 8 s, followed by a 2-s inter-trial-interval. Participants were instructed to look freely at the matrix until it disappeared. A 2-min break was introduced between blocks to reduce fatigue. Each block preceded by a 5-point eye-tracking calibration followed by a 5-point validation procedure.

**Figure 1:**
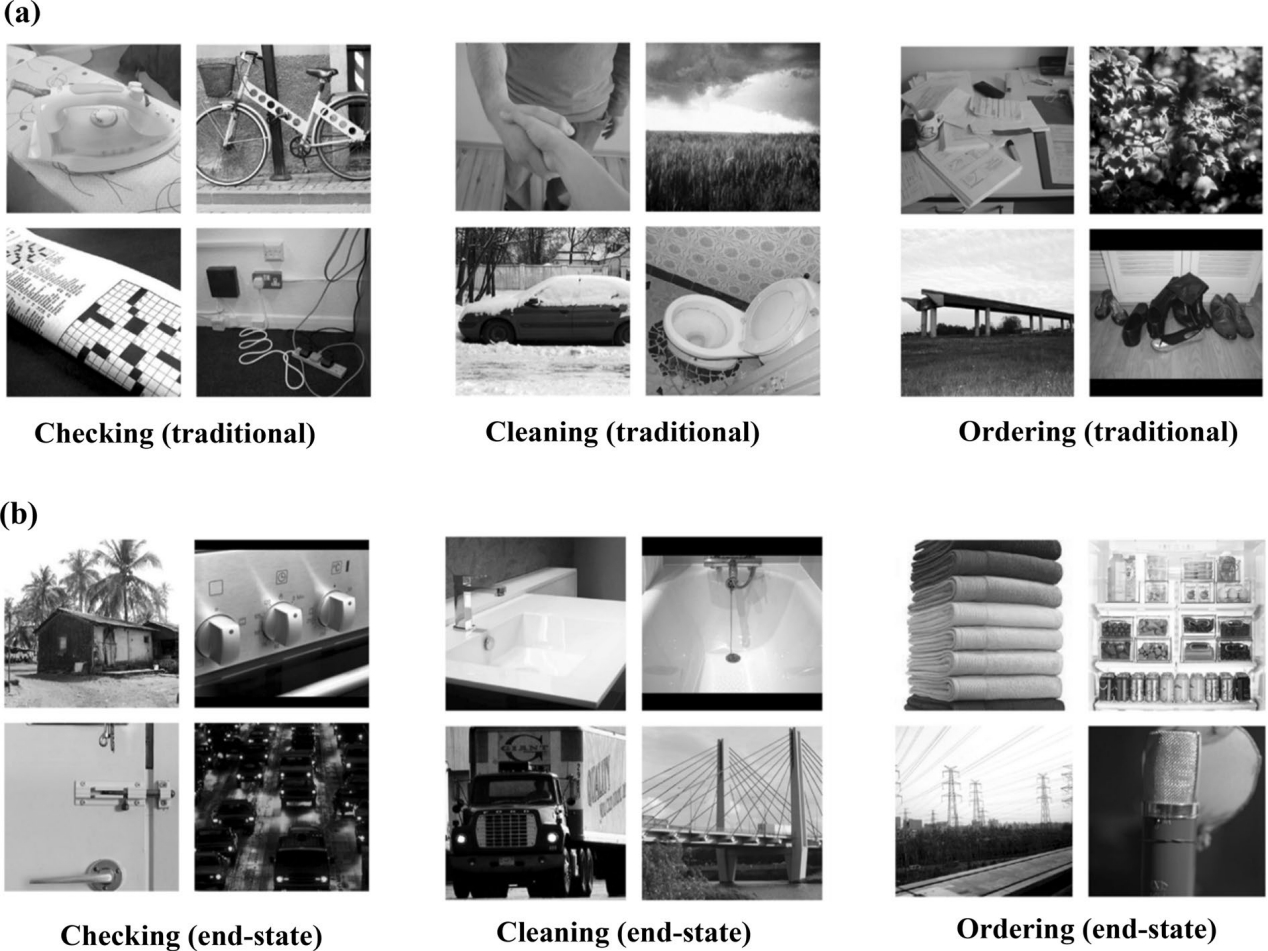
An example of a single matrix for the (**a**) traditional stimuli block; and (**b**) end-state stimuli block [Checking block (left), cleaning block (middle); ordering block (right)]. In each matrix each type of stimuli comprises a separate area of interest (AOI).

The study included two versions of the task described above. One version contrasted neutral pictures with pictures evoking obsession-related anxiety/discomfort, such as a dirty sink. We termed this version the "traditional" version, as it presented traditional OCD-related threat pictures (see Fig. 1a for an example of a single matrix, per block). The alternative version, which we termed the "end-state" version, contrasted neutral pictures (different from the ones used in the three blocks of the traditional version, again, to eliminate familiarity effects) with OCD-related stimuli depicting end-states of compulsive behaviors, such as a spotless sink (see Fig. 1b for a matrix example per block). Each matrix consisted of four stimuli – two neutral pictures and two OCD-related pictures.

### Pictures used

Neutral pictures were taken from the International Affective Picture System (IAPS^50^) and the Nencki affective picture system by discrete emotional categories (NAPS BE^51^), both providing valence and arousal scores ranging from 1 (low) to 9 (high). We chose pictures rated between 4-to-6 on valence, reflecting neutral valence, coupled with low arousal levels, ranging between 2 and 4. The final 36 neutral pictures had a mean valence rating of 5.2, and a mean arousal score of 3.7. Traditional *threat OCD pictures* were chosen from two well-validated OCD-specific datasets – the Maudsley Obsessive–Compulsive Stimuli Set (MOCSS^52^) and the Berlin Obsessive Compulsive Disorder-Picture Set^53^. In total 36 stimuli were selected, consisting of 12 pictures per category (i.e. checking, cleaning, ordering). *End-states pictures* were assembled specifically for the present study, and retrieved from the internet. We aimed at finding pictures that would "mirror" the traditional picture set, namely, 36 OCD-relevant pictures but that signal end-states of compulsive behaviors (12 per category). For example, for a picture of a dirty sink, a matching image would be that of a shiny clean one, and a picture of a turned-on gas knob would be mirrored by a clearly visible “off” sign. First, a dataset of 42 pictures per OCD-type was prepared (for a total 126 pictures). Next, two psychologists with expertise in diagnosing and treating OCD rated each picture according to its relevance to checking, cleaning, and ordering. The final pictures chosen for each block were those rated as highly relevant to the specific OCD theme represented in the corresponding block, by both raters, while ensuring low scores on the two alternative themes.

### Discomfort rating

To assess subjective discomfort experienced while viewing the OCD-related pictures, a computerized questionnaire was prepared and administered following the eye-tracking task – the Subjective Discomfort Questionnaire (SDQ). Specifically, the OCD-related pictures were presented one by one, with experienced discomfort (i.e. “how much discomfort do you experience”) per picture assessed using a 100-mm Visual Analog Scale (VAS), anchored with “much discomfort” on the right side and “no discomfort” on the left. Participants were then asked to place a vertical mark that best described the way they feel while viewing the picture. The VAS score was measured in millimeters from the left anchor of the scale to the subject’s mark^54^. Scores ranged between 0 and 100, with higher scores indicating higher levels of experienced discomfort. Total SDQ score was computed separately for each picture type by averaging the corresponding 36 pictures, for a total score ranging from 0 to 100. Cronbach’s alpha was 0.97 for the traditional items, and 0.94 for the end-state SDQ.

For each participant we then computed a *discomfort difference score* to assess the difference in experienced discomfort between the two stimuli types, by subtracting the score of the end-state picture from the score of the traditional picture, such that higher scores denoted larger reductions in experienced discomfort.

### Eye-tracking measures

Fixations were defined as at least 100 ms of stable fixation within 1-degree visual angle. For each matrix we defined two Areas of Interest (AOI's) – a neutral AOI (i.e. the neutral pictures) and an OCD-related AOI. *Total dwell time* was calculated by summing the total fixation duration on each AOI across matrices (in seconds), reflecting sustained attention – the degree to which attention is held by a specific type of stimulus, once detected^24^. *First fixation latency* was calculated by averaging the latency to first fixations, in milliseconds, per AOI. *First fixation location* was measured by counting the number of times the first fixation was in each AOI. These two measures reflect facilitated detection, or *vigilance* (i.e. the ease or speed in which specific stimuli is detected). A greater proportion of first fixations on one type of stimuli over the other, or shorter latencies to first fixate on that type, are considered evidence of vigilance^24^.

### Eye tracking apparatus

Eye-tracking data was collected and recorded using the remote head-free high-speed EyeLink Portable-Duo apparatus and the Experiment Builder software (SR-research, Ottawa, Ontario, Canada). Participants were seated approximately 700 mm away from the screen. Real-time monocular eye-tracking data was recorded at 500 Hz, with a 1920X1080-pixel display resolution. Eye-tracking data was processed using EyeLink Data Viewer software (SR-research, Ottawa, Ontario, Canada).

### Procedure

Participants were tested individually in a quiet room at the university. After providing informed consent, they were seated in front of the eye-tracking apparatus and told that during this task they will be presented with different matrices of different stimuli, appearing one after the other. They were also informed that before the appearance of each matrix a fixation cross would be shown at the center of the screen, on which they should fixate to make the matrix itself appear. They were then presented with a demonstration of this contingency. Following this demonstration, participants were instructed to look freely at each matrix in any way they choose until it disappears, and the task commenced. Participants were randomized to completed either the traditional task version or the end-state version. Following the completion of the task participants were requested to fill out the corresponding SDQ.

A week following the first session participants returned to the lab and completed the task version they did not complete during the first session. Order of task versions was counterbalanced across participants. Following the completion of Session 2, participants filled out the questionnaires, and were then thanked and debriefed.

### Data analysis

A sample of 60 has a power of 80% to detect a Group-by-AOI (see above) interaction at an alpha level of 0.05, of an effect size similar to that reported in previous studies of attention allocation in OCD (ranging between 0.12 and 0.20^11,18^). Hence, 30 participants per group was determined as the target sample size for this study. Power analysis was performed using G*Power 3.1.9.4^55^. Independent samples t-tests compared between-groups descriptive characteristics, with a chi-square test used to compare groups on gender distribution.

To examine group differences on attention allocation as a unified process we first performed a 2-by-3-by-2-by-2 multivariate analysis of variance (MANOVA), with group (HOC, LOC) as a between-subjects factor, and Block (checking, cleaning, ordering), Condition (traditional, end-state), and AOI (OCD-related, neutral) as within-subject factors. Next, to examine group differences on the different eye-tracking variables of attention allocation, we performed a similar 2-by-3-by-2-by-2 repeated measures ANOVA for each dependent measure. To further explore the stability of emergent attention allocation patterns across time, we also conducted a time-course analysis by adding Epoch as a second within-subject variable (i.e. Epochs 1 to 4). Specifically, we divided each 8-s trial into four 2-s time epochs^15,56,57^.

To address OCD subtyping and related attentional allocation to corresponding stimuli types, we conducted an exploratory analysis using specific OCD-subtypes scores from the OCI-R as the grouping variable (see Supplementary Material for a detail description of group composition and criteria), rather than the OCI-R total score (for a similar data analyses plan, see^11^). Specifically, we explored the three main OCD subtypes, namely, cleaning, checking, and ordering (We did not explore the other OCD subtypes per the OCI-R – obsessing, neutralizing and hoarding). Accordingly, three separate repeated-measures ANOVAs were carried out, one per block, with Group (per sub-type; see below) as a between-subjects factor, and Condition (traditional, end-state), and AOI (OCD-related, neutral) as within-subject factors.

Reliability of the eye-tracking measures was assessed for three variants of the total dwell time measure, as done in previous studies using the same task^47^ – total dwell time on the OCD-related AOI; total dwell time on the neutral AOI; and percent dwell time on the OCD-related AOI (DT%). Internal consistency was examined using Cronbach’s α. This was done for the entire sample, and separately for each group, treating each trial (i.e. each matrix) as a single item. Finally, an independent-sample *t* test was used to examine group difference on discomfort difference scores.

As three independent variables were within-subject variables (i.e. Block, Condition, AOI), the MANOVA was conducted using the 'stats' package in R (version 4.3.1). All other statistical analyses were conducted using SPSS (IBM; version 25.0) and were 2-sided, using α of 0.05. Effect sizes are reported using p values for ANOVAs and Cohen’s d for mean comparisons. Bonferroni correction was applied to multiple comparisons.

## Results

### Demographic characteristics

Demographic and clinical characteristics of the groups are described in Table 1. Significant group differences were noted on all clinical measures, all *p*s < 0.001. No group differences emerged for age or gender distribution. (As hoarding is no longer considered an OCD symptom/subtype per DSM-5, we recalculated the OCI-R total score of each group without the three Hoarding items. The HOC group score still remained significantly higher than the LOC group score (HOC: *M* = 30.03, *SD* = 7.16, LOC: *M* = 6.77, *SD* = 4.23, *t*(55) = 14.71 *p* = .00, *Cohen’s d* = 3.90). Exploring the score range per group showed no overlap between groups (HOC: range = 19–46; LOC: range = 0–16)).

**Table 1:** Demographic and clinical characteristics of the two groups.

Measure	LOC group (n = 27)		HOC group (n = 30)	
	M	SD	M	SD
Age	23.67^a^	2.15	23.57^a^	1.30
Gender ratio (M:W)	7:20^a^	–	7:23^a^	–
OCI-R				
Total score	8.74^a^	5.39	35.43^b^	8.18
Subscale scores				
Washing	0.74^a^	1.13	5.17^b^	2.76
Obsessing	1.48^a^	1.67	6.57^b^	2.73
Hoarding	1.96^a^	1.58	5.40^b^	2.50
Ordering	2.04^a^	1.89	7.73^b^	1.85
Checking	1.78^a^	1.69	7.10^b^	2.72
Neutralizing	0.74^a^	0.94	3.47^b^	2.95
DASS-21				
Depression	0.96^a^	1.16	4.83^b^	5.20
Anxiety	0.70^a^	0.86	5.00^b^	4.15
Stress	2.26^a^	2.06	9.23^b^	5.58

Different superscripts signify differences between groups at *p* < 0.001. *LOC* low obsessive–compulsive tendencies, *HOC* high obsessive–compulsive tendencies, *OCI*-*R* obsessive–compulsive inventory-revised, *DASS*-*21* depression, anxiety and stress scales-21.

### Eye-tracking measures

The Group × Condition × AOI × Block interaction was not significant* F*(3, 605) = 1.07, *p* = 0.37. However, a significant Group × Condition × AOI emerged, *F*(3, 605) = 5.46, *p* < 0.001, affording the exploration of specific dependent variables via separate three ANOVAs.

### Sustained allocation (total dwell time)

The omnibus Group (HOC, LOC) × Block (checking, cleaning, ordering) × Condition (traditional, end-state) × AOI (OCD-related, neutral) interaction was not significant* F*(2, 54) = 3.06, *p* = 0.17. However, a significant Group × Condition × AOI emerged, *F*(1, 55) = 4.89, *p* = 0.03, *η*^2^_p_ = 0.08, indicating differential dwell time patterns of the two groups for the OCD-related and the neutral AOIs, across the two conditions. We therefore collapsed across blocks for the remaining analyses. This interaction remained significant after introducing depression, anxiety and stress scores as covariates, *F*(1,52) = 4.10, *p* = 0.048, *η*^2^_p_ = 0.07.

**Figure 2:**
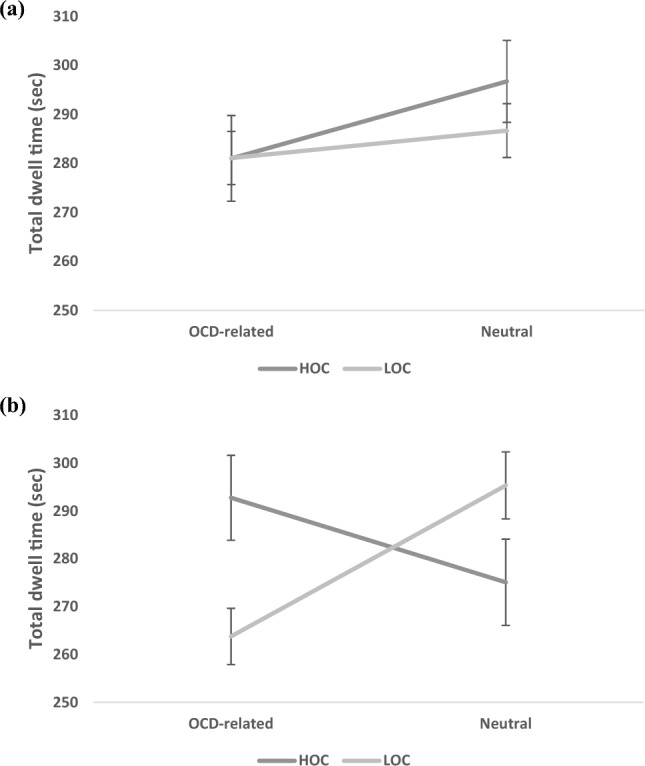
Total dwell time (in seconds) by Area of interest (AOI) and Group collapsed across blocks: (**a**) the traditional stimuli condition; and (**b**) the end-state stimuli condition. Error bars denote standard error of the mean. *OCD* obsessive–compulsive disorder, *HOC* high obsessive–compulsive tendencies, *LOC* low obsessive–compulsive tendencies.

Follow-up analyses of the Group × AOI interaction per Condition (traditional, end-state), revealed a significant interaction in the end-state condition, *F*(1, 55) = 4.50, *p* = 0.04, *η*^2^_p_ = 0.08, but not in the traditional condition, *F*(1, 55) = 0.41, *p* = 0.52 (see Fig. 2a and b for the traditional and end-state conditions, respectively).

Simple effects analyses for the end-state condition using independent t-tests showed that the HOC group spent significantly more time fixating on the OCD-related AOI (*M* = 291.82, SD = 47.27), compared with the LOC group (*M* = 263.71, SD = 31.74), *t*(55) = 2.60 *p* = 0.01, *Cohen’s d* = 0.69. No significant differences were found for the neutral AOI, *t*(55) = 1.32 *p* = 0.19. Exploring the stability of the Group-by-AOI interaction across time of the end-state task version showed no Epoch-related significant results. Specifically, the Group-by-Block-by-AOI-by-Epoch, and the Group-by-AOI-by-Epoch interaction effects were not significant, *F*(6, 50) = 0.47, *p* = 0.82, and *F*(3, 53) = 1.16, *p* = 0.33, respectively. These non-significant Epoch-related effects reflect a consistent pattern of attention allocation across stimuli presentation.

Internal consistency for total dwell time on each AOI, and for DT%, were high for the full sample, ranging from 0.83 to 0.89, and within groups, ranging from 0.74 to 0.93 (See Table 2 for full results).

**Table 2:** Internal consistency.

	Full sample	LOC group	HOC group
	(n = 57)	(n = 27)	(n = 30)
OCD-related traditional task			
Total DT – neutral stimuli	0.89	0.82	0.91
Total DT – OCD-related stimuli	0.83	0.74	0.86
DT% – OCD-related stimuli	0.87	0.75	0.91
OCD-related end-state task			
Total DT – neutral stimuli	0.84	0.89	0.79
Total DT – OCD-related stimuli	0.84	0.81	0.85
DT% – OCD-related stimuli	0.89	0.79	0.93

*DT* dwell time, DT% = percentage of dwell time spent on OCD-related stimuli out of the total dwell time spent on both the OCD-related and the neutral stimuli.

While the Group × Block × Condition × AOI was not significant when grouping participants based on total OCI-R scores, we conducted an exploratory within-block analyses using specific OCD-subtypes scores from the OCI-R as the grouping variable (for a similar data analyses approach, see^11^. Our exploratory within-block analyses revealed a significant Group × Condition × AOI interaction effect only for the cleaning block. These results are reported in full and discussed in the online Supplementary Material.

### Vigilance (first fixation measures)

For first fixation location, the omnibus Group (HOC, LOC) × Block (checking, cleaning, ordering) × Condition (traditional, end-state) × AOI (OCD-related, neutral) interaction was not significant* F*(2, 54) = 0.45, *p* = 0.64, as well as the Group × Condition × AOI interaction, *F*(1, 55) = 1.29, *p* = 0.26. Similarly, for latency to first fixation, the omnibus interaction was not significant* F*(2, 54) = 1.07, *p* = 0.35, as was the Group × Condition × AOI interaction, *F*(1, 55) = 1.32, *p* = 0.25.

### Discomfort difference score

A significant group difference emerged,* t*(53) = 4.67, *p* < 0.001, *Cohen’s d* = 1.26, showing a higher mean difference in subjective discomfort scores in the HOC group, *M*_HOC_ = 40.05, SD = 12.65, compared to the LOC group, *M*_LOC_ = 24.07, SD = 12.68. Exploring subjective discomfort differences separately for each block showed similar results – checking block: *M*_HOC_ = 23.53, SD = 12.63, *M*_LOC_ = 14.40, SD = 14.21, *t*(53) = 2.51, *p* = 0.01, *Cohen’s d* = 0.67; cleaning block: *M*_HOC_ = 48.89, SD = 16.40, *M*_LOC_ = 35.14, SD = 16.99,* t*(53) = 3.05, *p* = 0.004, *Cohen’s d* = 0.82; ordering block: *M*_HOC_ = 46.02, SD = 21.40, *M*_LOC_ = 22.67, SD = 13.80, *t*(53) = 4.85, *p* < 0.001, *Cohen’s d* = 1.29.

## Discussion

This is the first study to date to examine attention allocation to two types of OCD-related stimuli, compared to neutral ones – traditional threat stimuli (assumed to provoke related obsessions) and end-state stimuli (depicting end-states of compulsions). Specifically, gaze patterns of participants with high and low levels of OC symptoms were assessed and compared while freely viewing different matrices comprised of OCD-related and neutral pictures. While no differences in attention allocation patterns emerged in the traditional task version, results showed that HOC participants, compared to LOC participants, spent more time fixating on end-state stimuli. The task exhibited good-to-excellent psychometric properties, across and within groups, increasing our confidence in obtained results.

The traditional task version yielded no significant findings – not for first fixation measures of vigilance (first fixation location and latency), nor for attentional maintenance (total dwell time). While the lack of evidence for vigilance is in line with previous studies in the field^16^, the lack of evidence for a maintenance bias is divergent from most prior studies^16^. What may explain this divergence of results? One possible explanation may be related to the complexity of the visual display used (i.e. number of co-presented stimuli) – while most extant studies presented only two stimuli at once, the current study used more complex visual displays containing four co-presented stimuli in each trial. The fact that the only other study that failed to find evidence for attentional maintenance in OCD is also the only one that used displays of four co-presented stimuli^15^ further strengthen this interpretation. This explanation is also in line with attentional research suggesting that eye-tracking measures are affected by the number of presented stimuli, such that gaze differences are more readily detected when participants are presented with simple and limited visual displays^26,27^. Still, more research using more complex displays as used in the present study is now needed to further validate present findings. A second possible reason may be that the traditional stimuli were also more salient for the non-OC participants due to their visual nature and/or content, thereby capturing their attention. For example, while a picture of a filthy toilet may be threatening for OC individuals, it may also be more salient for non-OC individuals (compared with neutral pictures), limiting the possibility to detect group differences in attention allocation. Yet, as the traditional threat stimuli were taken from two validated OCD-specific datasets^52,53^, widely-used in previous studies in the field which showed group differences in attentional maintenance^16^, makes this latter possibility, in our view, less likely.

Unlike the traditional task, significant evidence for a maintenance bias emerged in the end-state version, with HOC participants dwelling longer on OCD-related stimuli, compared with LOC participants. Results also showed this attention allocation pattern to be stable across time. Two possible explanations may underlie this results pattern – increased threat sensitivity and/or reward-related processes. According to first, end-state stimuli may have evoked obsession-related distress among OC participants, as these stimuli are still OCD-relevant (even if to a lesser extent than the traditional ones), thereby capturing their attention^11,18^. Thus, a clean spotless sink may still have instigated related obsessions about contamination among OC participants. Conversely, for non-OC participants, unlike the traditional stimuli, end-state stimuli have no increased saliency. This possibility is in line, for example, with research in OCD that use “end-states” stimuli (e.g. a turned-off gas stove) to explore OCD-related checking compulsions^58,59^. Relatedly, while end-states stimuli may indeed signal the completion of the compulsive act, they may still then trigger subsequent obsessions and/or the need to perform another compulsive act. Clinical experience shows that when performing compulsions (e.g. compulsively cleaning a toilet), the end of one compulsion may become a trigger for the next compulsion (e.g. a clean toilet becomes a visual trigger for further cleaning the toilet) creating a series of compulsive acts^60–63^. According to the second possibility, attentional maintenance may reflect the relief “brought on” by end-states stimuli, capturing OC participants’ attention due to their (negatively-reinforced) rewarding nature. Relatedly, present result also showed a higher discomfort difference score among HOC, compared with LOC participants, reflecting greater experienced relief. This possibility is in line with the phenomenology of compulsive behavior, which is believed to be perpetuated due to negative reinforcement processes^64^. It also corresponds with recent research showing alterations in reward functioning in OCD^65,66^, and with attentional research demonstrating an association between OCD symptoms and difficulty disengaging attention from appetitive images^67,68^. Hence, from this perspective, it is possible that HOC individuals dwelled longer on end-state stimuli due to their (negatively-reinforced) rewarding nature, and not only due to threat-related processes. Importantly, however, as this is the first study to examine attention allocation toward end-state OCD-related stimuli, current conceptualizations of emergent results should be taken with caution, and more research is now needed to tease apart these two possibilities.

Taken together, present findings (i.e. lack of group differences on the traditional task coupled with group differences on the end-state task) may be also viewed via the "inference-based approach" (IBA) of OCD. According to this model, obsession arise from a reasoning narrative with no direct support from sensory-based information, also called "inferential confusion”^69–73^. In support of the IBA of OCD, research has shown inferential confusion to predict OCD symptoms^73–75^. For example, Audet et al.^75^ presented participants with a series of scenarios, some with and some without direct evidence for an actual reason for a potential intrusive thought. Following each scenario, participants were asked to choose potential intrusions they might experience due to the scenario. Results showed that participants’ reactions to OCD specific scenarios were more related to OCD symptoms when these were not supported by direct evidence, compared to when they were^76^. Viewed from this perspective, a possible explanation for current results may be related to the difference between the two stimulus types in the “direct evidence” they afford – while traditional OCD-related threat stimuli (e.g. a clearly visible dirty sink) provide direct evidence for “contamination”, the end-states stimuli (e.g. a spotless shining sink) do not. Moreover, from this viewpoint, the end-state stimuli may be more ecological-valid compared with traditional OCD-related threat stimuli used in previous attentional research in the field (e.g.^11,18^).

This study has several limitations. First, the study examined a sample of participants with high levels of OC symptoms, rather than a clinical OCD sample. Still, we used a high cutoff score of 27 on the OCI-R, reflecting severe OCD^38^, when recruiting participants. Also, OCI-R scores were assessed twice, once during initial participant screening, and once on the day of study participation, to verify score stability. Using samples of high and low scorers on measures of OCD has been shown to be relevant to the understanding of the disorder (for a review see^77^), and was proven useful in previous research conducted in our laboratory, in which results of HOC vs LOC participants were later successfully replicated in clinical samples^40,41^. Finally, subclinical OCD (i.e. individuals with high OCD symptoms) is related to significant impairments in various life domains, similar to those observed among OCD patients, conceptualized as a risk factor for later development of clinical OCD^6^. Yet, future studies should replicate the present one among clinically diagnosed OCD patients. Second, the end-state stimuli set was prepared and validated specifically for the purpose of the current study, not chosen from established stimuli datasets, as were the traditional OCD stimuli. Designated validation studies of potentially OCD stimuli signaling end-states, specifically via the two-by-two picture matrices, are now needed to enable further exploring the effects of these stimuli on attention allocation patterns. Moreover, as noted above, present findings showing sustained attention to end-states stimuli could stem from traditional anxiety-related processes, from (negative-reinforcement) reward-related processes^64,78^, or a combination of both. Future research could assess the subjective experience of participants to presented pictures beyond mere discomfort (i.e. pleasure). Third, as this was the first study to explore attention allocation to both types of stimuli, we opted to explore each separately using two separate task versions. Future studies may wish to mix the two types within a single task. Fourth, while trying to address the large heterogeneity of OCD by including stimuli related to major OCD dimensions (i.e. contamination, checking, and symmetry/order^52^), and through our exploratory analyses, future research could better address this issue by either including a wider variety of symptom-to-stimulus types (i.e. using ideographically tailored stimuli), or by including a narrower sample, limited to specific OCD themes/subtypes. Including additional OCD-relevant symptom-to-stimulus types (such as pictures provoking aggressive, sexual repugnant or autonomous obsessions among corresponding samples) may enhance the generalizability of current findings, as specific subtypes may exhibit different attentional allocation patterns to these stimuli types (e.g. avoiding images of blood or knives). Finally, in the present study, OCD-related stimuli were presented alongside neutral stimuli. Contrasting the two stimuli types more directly may yield a different results pattern, as this may create a more direct competition over one’s attention, while eliminating the option to "escape" to non-OCD neutral stimuli^54^.

Current findings provide preliminary evidence for attentional biases to OCD-related stimuli signaling compulsions end-states, a possibility that has been mainly overlooked in extant attentional research in OCD. These findings may further suggest to incorporate end-state OCD content into extant attention bias modification trainings (ABMT) procedures in OCD, which, to date, only include OCD-related threat stimuli, especially as these mostly show a reduction in attention bias with no corresponding reduction in symptoms^79–81^. Current findings may also provide some support for the need to include end-state “triggers” in exposure and response prevention (ERP) techniques for treating OCD. If end-states-related cues/situations also trigger OCD-related symptoms, due to, among others, inferential confusion, uncertainty and doubt experiences^60,63,72,82^, including these in ERP may enhance its efficacy.

## Supplementary Information

Supplementary Figures.

Supplementary Information.

## Data Availability

Data are openly available in Open Science Foundation at https://osf.io/mkwpx/?view_only=73ac1f835bf44d0d86d601652da04fd3.

## References

[1] Ruscio AM, Stein DJ, Chiu WT, Kessler RC (2010). The epidemiology of obsessive-compulsive disorder in the National Comorbidity Survey Replication. Mol. Psychiatry.

[2] Association, A. P. & Association, A. P. Diagnostic and statistical manual of mental disorders: DSM-5 (2013).

[3] Grabe HJ (2001). Lifetime-comorbidity of obsessive-compulsive disorder and subclinical obsessive-compulsive disorder in Northern Germany. Eur. Arch. Psychiatry Clin. Neurosci..

[4] Mataix-Cols D, Vallejo J, Sanchez-Turet M (2000). The cut-off point in sub-clinical obsessive-compulsive research. Behav. Cogn. Psychother..

[5] Welkowitz LA, Struening EL, Pittman J, Guardino M, Welkowitz J (2000). Obsessive-compulsive disorder and comorbid anxiety problems in a national anxiety screening sample. J. Anxiety Disord..

[6] Fullana MA (2009). Obsessions and compulsions in the community: Prevalence, interference, help-seeking, developmental stability, and co-occurring psychiatric conditions. Am. J. Psychiatry.

[7] Salkovskis PM, Forrester E, Richards C (1998). Cognitive–behavioural approach to understanding obsessional thinking. Br. J. Psychiatry.

[8] Salkovskis P, Shafran R, Rachman S, Freeston MH (1999). Multiple pathways to inflated responsibility beliefs in obsessional problems: Possible origins and implications for therapy and research. Behav. Res. Ther..

[9] Pleva J, Wade TD (2006). The mediating effects of misinterpretation of intrusive thoughts on obsessive-compulsive symptoms. Behav. Res. Ther..

[10] Cohen Y, Lachenmeyer JR, Springer C (2003). Anxiety and selective attention in obsessive–compulsive disorder. Behav. Res. Ther..

[11] Cludius B, Wenzlaff F, Briken P, Wittekind CE (2019). Attentional biases of vigilance and maintenance in obsessive-compulsive disorder: An eye-tracking study. J. Obs. Compuls. Relat. Disord..

[12] Salkovskis PM, McGuire J, Salkovskis PM, McGuire J (2003). Cognitive-behavioural theory of OCD. Obsessive-Compulsive Disorder: Theory, Research and Treatment.

[13] Lazarov A (2019). Attention to threat in posttraumatic stress disorder as indexed by eye-tracking indices: A systematic review. Psychol. Med..

[14] Armstrong T, Olatunji BO, Sarawgi S, Simmons C (2010). Orienting and maintenance of gaze in contamination fear: Biases for disgust and fear cues. Behav. Res. Ther..

[15] Armstrong T, Sarawgi S, Olatunji BO (2012). Attentional bias toward threat in contamination fear: Overt components and behavioral correlates. J. Abnorm. Psychol..

[16] Basel D, Hallel H, Dar R, Lazarov A (2023). Attention allocation in OCD: A systematic review and meta-analysis of eye-tracking-based research. J. Affect. Disord..

[17] Bradley MC (2016). Obsessive–compulsive symptoms and attentional bias: An eye-tracking methodology. J. Behav. Ther. Exp. Psychiatry.

[18] Mullen M (2021). Attentional bias in individuals with obsessive-compulsive disorder: A preliminary eye-tracking study. J. Behav. Cogn. Ther..

[19] Lazarov A (2021). Increased attention allocation to socially threatening faces in social anxiety disorder: A replication study. J. Affect. Disord..

[20] Lilienfeld SO, Strother AN (2020). Psychological measurement and the replication crisis: Four sacred cows. Can. Psychol. Psychol. Can..

[21] McNally RJ (2019). Attentional bias for threat: Crisis or opportunity?. Clin. Psychol. Rev..

[22] Parsons S, Kruijt A-W, Fox E (2019). Psychological science needs a standard practice of reporting the reliability of cognitive-behavioral measurements. Adv. Methods Pract. Psychol. Sci..

[23] Skinner IW (2018). The reliability of eyetracking to assess attentional bias to threatening words in healthy individuals. Behav. Res. Methods.

[24] Waechter S, Nelson AL, Wright C, Hyatt A, Oakman J (2014). Measuring attentional bias to threat: Reliability of dot probe and eye movement indices. Cogn. Ther. Res..

[25] Lazarov A, Abend R, Bar-Haim Y (2016). Social anxiety is related to increased dwell time on socially threatening faces. J. Affect. Disord..

[26] Richards HJ, Benson V, Donnelly N, Hadwin JA (2014). Exploring the function of selective attention and hypervigilance for threat in anxiety. Clin. Psychol. Rev..

[27] Yates A, Ashwin C, Fox E (2010). Does emotion processing require attention? The effects of fear conditioning and perceptual load. Emotion.

[28] Denys D (2011). Obsessionality & compulsivity: A phenomenology of obsessive-compulsive disorder. Philos. Ethics Humanit. Med..

[29] Grant JE (2014). Obsessive–compulsive disorder. N. Engl. J. Med..

[30] Kwak S-M, Na DL, Kim G, Kim GS, Lee J-H (2006). Use of eye movement to measure smokers' attentional bias to smoking-related cues. Cyberpsychol. Behav..

[31] McGrath DS, Sears CR, Fernandez A, Dobson KS (2021). Attentional biases in low-risk and high-risk gamblers and the moderating effect of daily psychosocial stress. Addict. Res. Theory.

[32] Parvaz MA (2021). Attention bias modification in drug addiction: Enhancing control of subsequent habits. Proc. Natl. Acad. Sci..

[33] Soleymani A, Ivanov Y, Mathot S, de Jong PJ (2020). Free-viewing multi-stimulus eye tracking task to index attention bias for alcohol versus soda cues: Satisfactory reliability and criterion validity. Addict. Behav..

[34] Koob GF (2013). Negative reinforcement in drug addiction: The darkness within. Curr. Opin. Neurobiol..

[35] Ahmed SH, Koob GF (2005). Transition to drug addiction: A negative reinforcement model based on an allostatic decrease in reward function. Psychopharmacology.

[36] Baker TB, Piper ME, McCarthy DE, Majeskie MR, Fiore MC (2004). Addiction motivation reformulated: An affective processing model of negative reinforcement. Psychol. Rev..

[37] Foa EB (2002). The obsessive-compulsive inventory: Development and validation of a short version. Psychol. Assess..

[38] Abramovitch A, Abramowitz JS, Riemann BC, McKay D (2020). Severity benchmarks and contemporary clinical norms for the Obsessive-Compulsive Inventory-Revised (OCI-R). J. Obs. Compuls. Relat. Disord..

[39] Lovibond PF, Lovibond SH (1995). The structure of negative emotional states: Comparison of the Depression Anxiety Stress Scales (DASS) with the Beck Depression and Anxiety Inventories. Behav. Res. Ther..

[40] Lazarov A, Liberman N, Hermesh H, Dar R (2014). Seeking proxies for internal states in obsessive–compulsive disorder. J. Abnorm. Psychol..

[41] Lazarov A (2021). Attenuated access to emotions in obsessive-compulsive disorder. Behav. Ther..

[42] Hajcak G, Huppert JD, Simons RF, Foa EB (2004). Psychometric properties of the OCI-R in a college sample. Behav. Res. Ther..

[43] Lazarov A, Friedman A, Comay O, Liberman N, Dar R (2020). Obsessive-compulsive symptoms are related to reduced awareness of emotional valence. J. Affect. Disord..

[44] Antony MM, Bieling PJ, Cox BJ, Enns MW, Swinson RP (1998). Psychometric properties of the 42-item and 21-item versions of the Depression Anxiety Stress Scales in clinical groups and a community sample. Psychol. Assess..

[45] Henry JD, Crawford JR (2005). The short-form version of the Depression Anxiety Stress Scales (DASS-21): Construct validity and normative data in a large non-clinical sample. Br. J. Clin. Psychol..

[46] Lovibond PF (1998). Long-term stability of depression, anxiety, and stress syndromes. J. Abnorm. Psychol..

[47] Lazarov A (2021). Attention allocation in posttraumatic stress disorder: An eye-tracking study. Psychol. Med..

[48] Suarez-Jimenez B (2022). Attention allocation to negatively-valenced stimuli in PTSD is associated with reward-related neural pathways. Psychol. Med..

[49] Clark DA (2019). Cognitive-Behavioral Therapy for OCD and its Subtypes.

[50] Lang, P. J., Bradley, M. M. & Cuthbert, B. N. International affective picture system (IAPS): Instruction manual and affective ratings. *The center for research in psychophysiology, University of Florida* (1999).

[51] Riegel M (2016). Characterization of the Nencki affective picture system by discrete emotional categories (NAPS BE). Behav. Res. Methods.

[52] Mataix-Cols D, Lawrence NS, Wooderson S, Speckens A, Phillips ML (2009). The Maudsley obsessive-compulsive stimuli set: Validation of a standardized paradigm for symptom-specific provocation in obsessive–compulsive disorder. Psychiatry Res..

[53] Simon D, Kischkel E, Spielberg R, Kathmann N (2012). A pilot study on the validity of using pictures and videos for individualized symptom provocation in obsessive–compulsive disorder. Psychiatry Res..

[54] Elias S, Massad R, Lazarov A (2021). Visual attention patterns of socially anxious individuals when using facebook: An eye tracking study. Behav. Ther..

[55] Faul F, Erdfelder E, Lang A-G, Buchner A (2007). G* power 3: A flexible statistical power analysis program for the social, behavioral, and biomedical sciences. Behav. Res. Methods.

[56] Kimble MO, Fleming K, Bandy C, Kim J, Zambetti A (2010). Eye tracking and visual attention to threating stimuli in veterans of the Iraq war. J. Anxiety Disord..

[57] Felmingham KL, Rennie C, Manor B, Bryant RA (2011). Eye tracking and physiological reactivity to threatening stimuli in posttraumatic stress disorder. J. Anxiety Disord..

[58] van den Hout MA, Engelhard IM, de Boer C, du Bois A, Dek E (2008). Perseverative and compulsive-like staring causes uncertainty about perception. Behav. Res. Ther..

[59] van den Hout MA (2009). Uncertainty about perception and dissociation after compulsive-like staring: Time course of effects. Behav. Res. Ther..

[60] Rachman S (2002). A cognitive theory of compulsive checking. Behav. Res. Ther..

[61] Tolin DF (2001). Memory and memory confidence in obsessive–compulsive disorder. Behav. Res. Ther..

[62] Salkovskis PM, Forrester E (2002). Cognitive Approaches to Obsessions and Compulsions.

[63] van den Hout M, Kindt M (2003). Phenomenological validity of an OCD-memory model and the remember/know distinction. Behav. Res. Ther..

[64] Figee M (2011). Dysfunctional reward circuitry in obsessive-compulsive disorder. Biol. Psychiatry.

[65] Ferreira GM, Yücel M, Dawson A, Lorenzetti V, Fontenelle LF (2017). Investigating the role of anticipatory reward and habit strength in obsessive-compulsive disorder. CNS Spectr..

[66] Rouhani N (2019). Impaired generalization of reward but not loss in obsessive–compulsive disorder. Depress. Anxiety.

[67] Olatunji BO, Ciesielski BG, Zald DH (2011). A selective impairment in attentional disengagement from erotica in obsessive–compulsive disorder. Prog. Neuropsychopharmacol. Biol. Psychiatry.

[68] Olatunji BO (2021). Emotional induced attentional blink in obsessive-compulsive disorder. J. Affect. Disord..

[69] Aardema F, O’Connor K (2003). Seeing white bears that are not there: Inference processes in obsessions. J. Cogn. Psychother..

[70] Aardema F, O’Connor K (2007). The menace within: Obsessions and the self. J. Cogn. Psychother..

[71] Aardema F, O'Connor K (2012). Dissolving the tenacity of obsessional doubt: Implications for treatment outcome. J. Behav. Ther. Exp. Psychiatry.

[72] Aardema F, O’Connor KP, Emmelkamp PM, Marchand A, Todorov C (2005). Inferential confusion in obsessive–compulsive disorder: The inferential confusion questionnaire. Behav. Res. Ther..

[73] Aardema F (2010). The expanded version of the inferential confusion questionnaire: Further development and validation in clinical and non-clinical samples. J. Psychopathol. Behav. Assess..

[74] Aardema F, O'Connor KP, Emmelkamp PM (2006). Inferential confusion and obsessive beliefs in obsessive-compulsive disorder. Cogn. Behav. Ther..

[75] Aardema F, Radomsky AS, O’Connor KP, Julien D (2008). Inferential confusion, obsessive beliefs and obsessive–compulsive symptoms: A multidimensional investigation of cognitive domains. Clin. Psychol. Psychother..

[76] Audet J-S, Wong SF, Radomsky AS, Aardema F (2020). Not all intrusions are created equal: The role of context, feared-self perceptions and inferential confusion in the occurrence of abnormal intrusions. J. Obs. Compuls. Relat. Disord..

[77] Abramowitz JS (2014). The relevance of analogue studies for understanding obsessions and compulsions. Clin. Psychol. Rev..

[78] Choi J-S (2012). Altered brain activity during reward anticipation in pathological gambling and obsessive-compulsive disorder. PLoS One.

[79] Habedank, I., Lennartz, S. J., Arslan, R. C. & Ertle, A. Online attention bias modification for obsessive-compulsive disorder: A randomized controlled trial (2017). 10.17605/OSF.IO/U7CVE

[80] Najmi S, Amir N (2010). The effect of attention training on a behavioral test of contamination fears in individuals with subclinical obsessive-compulsive symptoms. J. Abnorm. Psychol..

[81] Rouel M, Smith E (2018). Attentional bias and its modification in contamination OCD symptomatology. Cogn. Ther. Res..

[82] Dar R, Lazarov A, Liberman N (2021). Seeking proxies for internal states (SPIS): Towards a novel model of obsessive-compulsive disorder. Behav. Res. Ther..

