# Supplementary material for: Increased attention allocation to stimuli reflecting end-states of compulsive behaviors among obsessive compulsive individuals
Source: Sci Rep. 2023 Jul 27;13:12190. doi: 10.1038/s41598-023-39459-x (PMC10374647; doi:10.1038/s41598-023-39459-x)
Supplement: Supplementary file 1 — Supplementary Figures. [file 41598_2023_39459_MOESM1_ESM.docx]

**(a)** Cleaning block: traditional stimuli condition

**(b)** Cleaning block: end-state stimuli condition

**Figure S1.** Total dwell time (in seconds) by Area of interest (AOI) and Group for the cleaning block: (a) the traditional stimuli condition; and (b) the end-state stimuli condition. Error bars denote standard error of the mean.

*Note.* OCD; obsessive-compulsive disorder; HCS, high contamination symptoms; LOC, low obsessive-compulsive tendencies.

**(a)** Checking block: traditional stimuli condition

**(b)** Checking block: end-state stimuli condition

**Figure S2.** Total dwell time (in seconds) by Area of interest (AOI) and Group for the checking block: (a) the traditional stimuli condition; and (b) the end-state stimuli condition. Error bars denote standard error of the mean.

*Note.* OCD; obsessive-compulsive disorder; HCS, high checking symptoms; LOC, low obsessive-compulsive tendencies.

**(a)** Ordering block: traditional stimuli condition

**(b)** Ordering block: end-state stimuli condition

**Figure S3.** Total dwell time (in seconds) by Area of interest (AOI) and Group for the ordering block: (a) the traditional stimuli condition; and (b) the end-state stimuli condition. Error bars denote standard error of the mean.

*Note.* OCD; obsessive-compulsive disorder; HOS, high ordering symptoms; LOC, low obsessive-compulsive tendencies.
