# Supplementary material for: Increased attention allocation to stimuli reflecting end-states of compulsive behaviors among obsessive compulsive individuals
Source: Sci Rep. 2023 Jul 27;13:12190. doi: 10.1038/s41598-023-39459-x (PMC10374647; doi:10.1038/s41598-023-39459-x)
Supplement: Supplementary file 2 — Supplementary Information. [file 41598_2023_39459_MOESM2_ESM.docx]

**Supplementary Material**

**Exploratory within-block analyses**

**Data Analysis**

We conducted a 2-by-2-by-2 repeated measures Analysis of Variance (ANOVA) within each of the three blocks (checking, cleaning, ordering), with Condition (traditional, end-state) and AOI (OCD-related, neutral) as within-subject factors, and group as a between-subjects factor. Here, we used specific OCD-subtypes scores from the OCI-R as the participants grouping variable, rather than the OCI-R total score (for a similar data analyses plan, see^1^). Specifically, cut-off scores for each sub-scale were defined as one SD above a validated mean of each sub-scale^2^. Thus, participants who scored >21 on the total OCI-R score, coupled with a score ≥6 on the OCI-R checking subscale, were defined as high on checking; participants who scored >21 on the total OCI-R score and >5 on the OCI-R cleaning subscale were defined as high on contamination; and participants who scored >21 on the total OCI-R score and >8 on the OCI-R ordering subscale were defined as high on ordering. This resulted in a final sample of 21 participants high on checking symptoms, 17 participants high on cleaning symptoms, and 15 participants high on ordering symptoms. The original LOC group (n=27) served as the control group.

All statistical analyses were conducted using SPSS (IBM; version 25.0) and were 2-sided, using α of 0.05. Effect sizes are reported using p values for ANOVAs and Cohen’s d for mean comparisons. Bonferroni correction was applied to multiple comparisons.

**Results**

**Cleaning block**

A significant Group×Condition×AOI interaction emerged, *F*(1, 42)=11.99, *p*=.003, *η^2^_p_*=.22. Follow-up analysis per condition revealed a significant Group×AOI interaction for the traditional condition, *F*(1, 42)=5.16, *p*=.03, *η^2^_p_*=.11 and for the OCD-related end-state condition, *F*(1, 42)=6.10, *p*=.02, *η^2^_p_*=.13 (see Figure S1a for the traditional condition and S1b for the end-state condition). Follow-up Independent samples t-test for the traditional condition showed that the HCS group spent significantly more time fixating on neutral AOI (*M*=110.37, *SD*=20.64), compared with the LOC (*M*=95.40, *SD*=15.15), *t*(42)=2.70, *p*=.008, *Cohen’s d*=0.86. No significant differences were found for the OCD-related AOI, *t*(42)=1.59, *p*=.12. For the end-state condition, results showed that the HCS group spent significantly more time fixating on the OCD-related AOI (*M*=91.26, *SD*=20.89), compared with the LOC group (*M*=78.06, *SD*=12.47), *t*(42)=2.63, *p*=.01, *Cohen’s d*=0.82. No significant differences were found for the neutral AOI, *t*(42)=1.98, *p*=.05. No significant results emerged for any of the first fixation measures – first fixation location, *F*(1, 42)=1.45, *p*=.24, and latency to first fixation, *F*(1, 42)=.16, *p*=.07.

**Checking block**

No significant Group×Condition×AOI interaction was found for total dwell time, *F*(1, 46)=3.95, *p*=.16, *η^2^_p=_*.08. Examining first fixation measures showed no significant results for first fixation location, *F*(1, 46)=.83, *p*=.36, or for latency to first fixation, *F*(1, 46)=1.50, *p*=.22.

**Ordering block**

Examining total dwell time revealed no significant Group×Condition×AOI interaction, *F*(1, 40)=.13, *p*=.72. No significant results emerged for first fixation location, *F*(1, 40)=.04, *p*=.84, or latency to first fixation, *F*(1, 40)=2.32, *p*=.14.

**Discussion**

Our exploratory analysis examining specific OCD subtypes (participants and stimuli) yielded significant findings only in the cleaning block, as different attention allocation patterns emerged in the two task versions between participants with high vs low scores on the cleaning subscale. Specifically, while the first showed avoidance tendencies of OCD-related threat stimuli among participants with high cleaning scores, the latter revealed an opposite pattern, namely, increased attention allocation to OCD-related end-state stimuli, among participants high on cleaning. This avoidance tendency of OCD-related threat stimuli is in line with previous findings showing that participants with high contamination fears make shorter fixations on contamination-related stimuli relative to other image types^3^.

Dissimilar from the cleaning block, no significant Group-by-Condition-by-AOI emerged for the checking and the ordering blocks. Considering the checking block, this lack of findings may be related to the nature of the end-state checking stimuli used in the present study, which may have provoked checking urges at a similar level as the traditional checking stimuli. Put differently, it is possible that a picture of a locked door or an unlocked one provoked an urge to check whether they are “truly” closed. Hence, it may be that for the checking block there was no “actual” end-state condition. This possibility is in line with previous studies in the realm of checking that use similar “end-state” stimuli (e.g., a turned off gas stove or light bulb) to explore checking urges, showing increased ensuing checking behaviors and uncertainty among participants with OCD compared to control participants^4-6^. Considering the ordering block, the lack of group differences may be related to the general tendency of most people, both high and low on ordering tendencies, to prefer more symmetrical versions of a given stimuli, a proposition that has been widely established in past research^7,8^. Thus, future research could refine the checking and ordering stimuli used in the present study.

While providing initial interesting insights, a major limitation of the present exploratory analysis is the small sample size of the OCD sub-type groups. As OCD is a highly heterogeneous disorder, future research should use larger samples to examine attentional allocation toward symptom-specific stimuli among symptom-specific populations. This, in turn, would enable a deeper understating of the field.

**References**

1. Cludius, B., Wenzlaff, F., Briken, P. & Wittekind, C. E. Attentional biases of vigilance and maintenance in obsessive-compulsive disorder: an eye-tracking study. *Journal of Obsessive-Compulsive and Related Disorders* **20**, 30-38; https://doi.org/10.1016/j.jocrd.2017.12.007 (2019).

2 Hajcak, G., Huppert, J. D., Simons, R. F. & Foa, E. B. Psychometric properties of the OCI-R in a college sample. *Behaviour research and therapy* **42**, 115-123; <https://doi.org/10.1016/j.brat.2003.08.002> (2004).

3. Armstrong, T., Sarawgi, S. & Olatunji, B. O. Attentional bias toward threat in contamination fear: Overt components and behavioral correlates. *Journal of abnormal psychology* **121**, 232; [https://doi.org/10.1037/a0024453](https://psycnet.apa.org/doi/10.1037/a0024453) (2012).

4. van den Hout, M. & Kindt, M. Phenomenological validity of an OCD-memory model and the remember/know distinction. *Behaviour Research and Therapy* **41**, 369-378; <https://doi.org/10.1016/S0005-7967(02)00097-9> (2003).

5. van den Hout, M. A., Engelhard, I. M., de Boer, C., du Bois, A. & Dek, E. Perseverative and compulsive-like staring causes uncertainty about perception. *Behaviour Research and Therapy* **46**, 1300-1304; <https://doi.org/10.1016/j.brat.2008.09.002> (2008).

6. van den Hout, M. A. *et al.* Uncertainty about perception and dissociation after compulsive-like staring: Time course of effects. *Behaviour Research and Therapy* **47**, 535-539; <https://doi.org/10.1016/j.brat.2009.03.001> (2009).

7. Eisenman, R. Complexity-simplicity: I. Preference for symmetry and rejection of complexity. *Psychonomic Science* **8**, 169-170; <https://doi.org/10.3758/BF03331603> (1967).

8 Rhodes, G., Proffitt, F., Grady, J. M. & Sumich, A. Facial symmetry and the perception of beauty. *Psychonomic Bulletin & Review* **5**, 659-669; https://doi.org/10.3758/BF03208842 (1998).
